# Effects of Exogenous Plant Growth Regulator Abscisic Acid-Induced Resistance in Rice on the Expression of Vitellogenin mRNA in *Nilaparvata lugens* (Hemiptera: Delphacidae) Adult Females

**DOI:** 10.1093/jisesa/ieu075

**Published:** 2014-01-01

**Authors:** Jing-Lan Liu, Xiao Chen, Hong-Mei Zhang, Xia Yang, Andrew Wong

**Affiliations:** ^1^ School of Plant Protection, Yangzhou University, Yangzhou 225009, China; ^3^ Department of Entomology, The University of Arizona, Tucson, AZ

**Keywords:** abscisic acid, rice, *Nilaparvata lugens*, *Nlvg* mRNA expression, callose content

## Abstract

Recent study showed that exogenous abscisic acid (ABA) acts as a regulator of plant resistance. This study investigated average injury scale and callose contents of rice, and vitellogenin ( *Nlvg* ) mRNA expression in *Nilaparvata lugens* (Stål) (Hemiptera: Delphacidae) adult females after third instar nymphs fed on exogenous ABA-treated susceptible [Taichung Native one (TN1)] and moderately resistant (IR42) rice cultivars. The results showed that exogenous ABA significantly decreased average injury scale of rice and *Nlvg* mRNA expression in *N. lugens* adults compared with the control (without ABA spraying). *Nlvg* mRNA expression in *N. lugens* adults decreased significantly after third instar nymphs fed on ABA-treated (5, 20, and 40 mg/liter) TN1 for 1 and 2 d, and for IR42, after fed on ABA-treated (20 and 40 mg/liter) rice plants for 1 d and after fed on ABA-treated (5, 20, and 40 mg/liter) rice for 2 d decreased significantly. The callose contents showed no significant change for TN1, while for IR42, significantly increased in roots and sheathes after *N. lugens* infestation under ABA treatments (20 and 40 mg/liter) compared with the control. The decrease of *Nlvg* mRNA expression may be partially attributed to the increase of callose content of plants. The results provide a profile for concerning the effects of ABA-induced rice plants’ defenses on phloem-feeding insects.

When attacked by insects, plants can produce endogenous signal molecules, such as stress hormones, including jasmonic acid (JA), salicylic acid (SA), ethylene (ET), and abscisic acid (ABA), that regulate signal transduction cascades in plant cells, leading to the activation and modulation of defense-related genes ( Li et al. 2002 ). In the last few decades, we primarily focused on the regulating effect of JA, SA and ET on abiotic stress ( Koo and Howe 2009 , Chuang et al. 2010 , Thaler et al. 2010 ), and ignored the regulating effect of ABA. A recent study shows that ABA has a negative impact on the regulating effects of JA, SA, and ET signaling by activating defense genes ( Mialoundama et al. 2009 ). In the early period of biotic invasion, ABA defense systems can resist biotic invasion and do not need the JA and SA systems ( Ingle et al. 2006 ). Similarly, exogenous hormones are plant growth regulators, which have important effects on plant resistance to insects. For example, JA-induced rice resistance to *Nilaparvata lugens* (Stål) (Hemiptera: Delphacidae), an important pest of rice in China and other Asian countries, in addition reduced the longevity and egg hatchability of adult *N. lugens* as well as the percentage of nymphs surviving to maturity ( Senthil-Nathan et al. 2009 ). These results show that exogenous JA-induced systemic defenses in rice have a direct negative impact on *N. lugens* survivorship ( Senthil-Nathan et al. 2009 ); however, the effects of ABA application on insects have received little attention.

Vitellogenins (Vg) are precursors of the major egg storage protein, vitellin, in many oviparous animals. Vgs of insects are large molecules that are synthesized in the fat body in a process that involves substantial structural modifications (e.g., glycosylation, lipidation, phosphorylation, and proteolytic cleavage) of the nascent protein prior to their secretion and transport to the ovaries ( Tufail and Takeda 2008 ). Nutrimental and environmental conditions affect the rate of yolk protein synthesis and accumulation in insects prior to oviposition ( Shapiro and Ferkovich 2002 ). Callose is widespread in higher plants. With respect to its chemical structure, it is a linear *β* -1, 3-glucan with some 1,6-branches ( Aspinall and Kessler 1957 ). It was first recognized at the sieve plates of phloem elements, around pollen mother cells, in pollen grains and in pollen tubes, and it is also found during normal growth at the cell plate, plasmodesmatal canals, root hairs, and spiral thickenings in tracheids ( Stone and Clarke 1992 ). At present, many papers showed that callose can resist the penetration of pathogen and virus ( Asselbergha and Hofte 2007 ), but little is found in plant resistance to insects. *N. lugens* (Stål) (Hemiptera: Delphacidae) is a phloem-feeding insect, and in which produces less injury on plants and induces similar responses to plants like disease bacteria after its feeding ( Iriti and Faoro 2008 ). Hao et al. (2008) showed that *N. lugens* feeding induced callose deposition in the sieve tubes at the point where the stylet was inserted, so collose may be related to rice plants resistance to insects. However, the effects of ABA on insects have received little attention.

To explore the effects of exogenous ABA treatments on plant resistance to insects, we examined rice plants resistance to insects, callose contents in the roots and sheaths of rice, and the gene expression of vitellogenin ( *Nlvg* ) in brachypterous *N. lugens* adult females after third instar nymphs fed on the ABA-treated rice plants. Our objective was to determine the effects of exogenous ABA on rice plant resistance and *Nlvg* mRNA expression in brachypterous *N. lugens* adult females and to explore the mechanism of rice plant resistance to the phloem-feeding insect.

## Materials and Methods

### 

#### Rice Varieties and Insects

Two rice cultivars were used: Taichung Native one (TN1), which is susceptible to brown planthopper *N. lugens* (Stål), and IR42, which is moderately resistant to brown planthopper *N. lugens* (Stål). Seeds were sown in cement tanks at an experimental farm of Yangzhou University. Six-leaf seedlings were selected from the tanks, and the soil of the plant roots was washed with tap water. Details of the cultivation method are described in Yoshida et al. (1972) and Wu et al. (2003), the hydroponic solution was replaced once a week, and its pH value was adjusted daily to 5.0 with 1 M HCl or 1 M NaOH. After approximately 30 d in Espino hydroponic solution, rice plants at the tillering stage were sprayed with 10 ml of ABA (5, 20, and 40 mg/liter, respectively) using a Jacto sprayer (Maquinas Agricolas Jacto S.A., Pompeia, Brazil) equipped with a cone nozzle (1-mm-diameter orifice, pressure of 45 psi, flow rate of 300 ml/min) for four continuous days in the afternoon. The control plants at the same stage were sprayed with 0.5% Teepol according to Dong et al. (2009) , and the treated rice plants were used for the experiments. A laboratory strain of *N. lugens* that was originally obtained from the China National Rice Research Institute (CNRRI; Hangzhou, China) was reared in a greenhouse at an ecological laboratory at Yangzhou University. Thirty third instar nymphs and one rice plant with one of the above ABA treatments were placed in a plastic pot, and the root of the treated rice plant was packaged with wet cotton. The plastic pot was incubated at 26 ± 1°C and 70–80% humidity under a 16:8 (L:D) h photoperiod, and the experiment was replicated 10 times. After 1 or 2 d, the rice plants were collected to measure the callose contents, and *N. lugens* was also collected and raised on the control rice plants (without ABA spraying) until adult emergence. After the adult emerged, the females were separated, and 20 females per replication were used separately to measure *Nlvg* mRNA expression in brachypterous adult females before mating at 2 d (2-d-old virgin females).

#### Rice Plant Resistance (Average Injury Scale) of Rice Plants

Six-leaf seedlings selected from the tanks were transplanted into 16-cm-diameter plastic pots, with one plant per pot. After approximately 30 d, the rice plants were sprayed with 10 ml of ABA (5, 20, and 40 mg/liter) using a Jacto sprayer (Maquinas Agricolas Jacto S.A.) equipped with a cone nozzle (1-mm-diameter orifice, pressure of 45 psi, flow rate of 300 ml/min) for four continuous days in the afternoon, and control rice plants at the same stage were sprayed with 0.5% Teepol according to Dong et al. (2009) . The treated and control rice plants were used for the experiments. There were eight repetitions for each treatment, and there was one rice plant in each repetition. Ten third instar nymphs *N. lugens* were released on the above rice plants and caged immediately. When brachypterous *N. lugens* adults emerged (approximately 10 d after the release), average injury scale of the rice plants was assessed according to a 9-level evaluation method ( Table 1 ), modified from the screening method used to determine the resistance of rice cultivars ( Choi 1979 , Wu et al. 2001 ).

**Table 1.** Scale of plant injury symptoms

Scale	Scale symptom description
1	Slight injuries, few yellow pitches on leaf sheaths
3	Leaf sheaths slightly yellow
5	Leaf sheaths clearly yellow, reduced tillering
7	Leaf sheaths severely yellow, plant dwarfing, and severely reduced tillering
9	General withering

#### *Nlvg* mRNA Expression in Brachypterous *N. lugens* Adult Females

To examine the effect of ABA treatments on *Nlvg* mRNA expression in brachypterous *N. lugens* adult females prior to mating, each treatment and control was replicated three times. *Nlvg* mRNA expression in females was measured. Total RNA isolation and cDNA preparation and quantitative real-time reverse transcriptase polymerase chain reaction (qRT-PCR) were estimated by the method designed by Ge et al. (2010) and Liu et al. (2012) . Total RNA was isolated from 20 adult females using an SV Total Isolation System Kit (Promega Corporation, Madison, WI). The synthesis of first-strand cDNA was carried out according to the instructions for the PrimeScript RT reagent Kit (TaKaRa Biotechnology (Dalian) Co., Ltd., Dalian, China). Synthesis was performed in a 10-µl total reaction volume containing 0.5 µg total RNA, 0.5 µl PrimeScript RT Enzyme mix I, 0.5 µl Oligo dT Primer (50 µM), 2 µl random hexamers (100 µM), 2 µl 5× PrimeScript Buffer (for Real Time), X µl total RNA and the addition of RNase-free dH _2_ O up to10 µl. The cDNA reverse transcriptase was done with the following cycling regime: 37°C for 15 min, 85°C 5 s, and 4°C for 5 min.

mRNA levels were measured by qRT-PCR using a One-Step SYBR Premix Ex Taq II Kit (TaKaRa Biotechnology). A qRT-PCR was performed in a 20-µl total reaction volume containing 0.1 µg total RNA, 0.8 µl primer mix containing 10 µM each forward and reverse gene-specific primers, 0.4 µl ROX Reference Dye II (50×), 2 µl cDNAs, 10 µl SYBR Premix EX Taq II and 6 µl H _2_ O. Non-template reactions (replacing total RNA with H _2_ O) and minus reverse transcriptase controls (replacing PrimeScript RT Enzyme Mix with H _2_ O) were used as negative controls. The qRT-PCR was conducted with the following cycling regime: initial incubation of 50°C for 5 min and 95°C for 10 s; 40 cycles of 95°C for 5 s; 56°C for 40 s; and 72°C for 15 s. Standard curves were obtained using a 10-fold serial dilution of pooled total RNA from 20 individuals. β-actin (EU179846), which has been recognized as a suitable normalization gene by a northern blotting test, was used as an internal control ( Liu et al. 2012 ). mRNA levels of *Nlvg* (AB353856) was quantified in relation to the expression of β-actin. The primer pair of each gene was designed to amplify approximately 150 bp PCR products, which were verified by nucleotide sequencing. To avoid genomic DNA contamination, the specific primers for Vg was designed to span an intron region (2,545 bp). Only data that showed a good efficiency (≥85%) and high correlation coefficients (≥95%) were included in the analysis. Means and standard errors for each time point were obtained from the average of three independent sample sets. The gene-specific primers used for Vg and β-actin was as follows: Vg-F: GTGGCTCGTTCAAGGTT ATGG;

Vg-R: GCAATCTCTGGGTGCTGTTG;

β-F: TGGACTTCGAGCAGG AAATGG;

β-R: ACGTCGCACTTCAGATCGAG.

#### Callose Contents in Rice Plants

The extracting and testing of callose contents in the roots and sheaths of rice after third instar *N. lugens* nymphs fed for 2 d were estimated by the methods described by Shi and Hou (2004) and Jones et al. (2006) . The 0.5 g root tip or sheath sample was immediately immersed in 1 ml 98% (v/v) alcohol in a centrifugal tube for fixing for approximately 60 min. A straw was used to remove the alcohol, and 500 µl of 1 M NaOH was added. The sample was then ground using a small mortar, placed in a water bath at 80°C for 15 min and then cooled. The mixture was centrifuged at 1,000 × *g* for 10 min to extract the supernatant fluid. A mixture of 142 µl of aniline blue, 75 µl of 1 M HCl, and 210 µl of 1 M glycine/NaOH (buffer solution, pH 9.5) was added to 71 µl of the supernatant fluid. The mixture was placed in a water bath at 50°C water for 20 min and then cooled. OD values were detected with the Cary Eclipse fluorospectrophotometer (Varian Co., Palo Alto, CA). The callose contents of roots and sheaths of the rice plants were calculated in terms of the fresh weight.

#### Statistical Analysis

*Nlvg* mRNA expression in brachypterous *N. lugens* adult females was analyzed using three-way analysis of variance (ANOVA; rice cultivars, days after third instar nymphs fed on ABA-treated rice plants, and ABA concentrations), and callose contents were analyzed using ANOVA (rice cultivars, rice organ, and ABA concentrations), and all multiple comparisons of means were conducted using Fisher’s Protected Least Significant Difference test and the GLM procedure ( SPSS 2002 ).

## Results

### 

#### Average Injury Scale of Rice Plants Infested by *N. lugens* Third Instar Nymphs After ABA Treatments

**Fig. 1.**
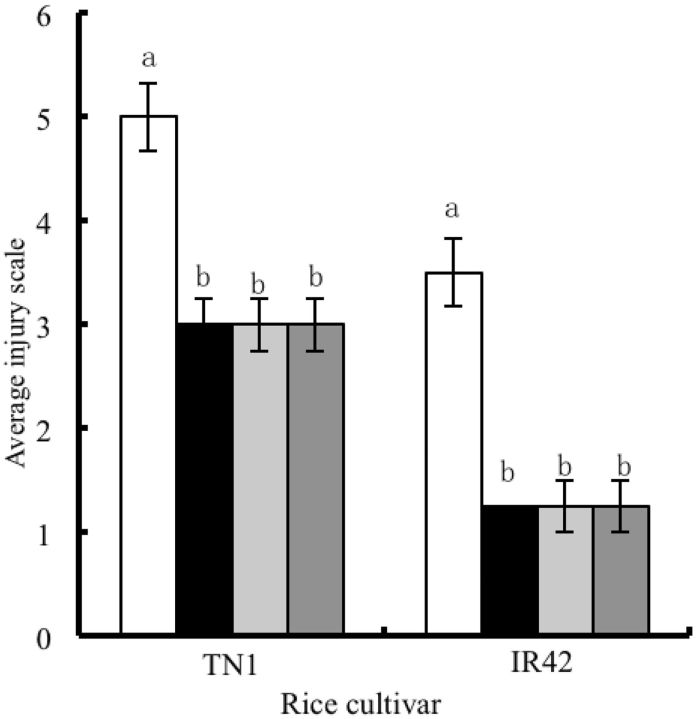
Effects of exogenous ABA treatments on the average injury scale of rice plants after infested by *N. lugens* . Data are presented as the means ± SE. Means followed by different letters indicate significant difference at the 5% level.

The average injury scale of ABA-treated TN1 ( *F* = 13.7, df = 3,28, *P* < 0.05) and IR42 ( *F* = 17.2, df = 3,28, *P* < 0.05) rice plants infested by *N. lugens* third instar nymphs decreased significantly compared with the control (0 mg/liter, without ABA spraying ; Fig. 1 ), indicating that ABA-treated rice plants are not suitable for *N. lugens* feeding or that ABA increased rice plant resistance to *N. lugens* .

#### *Nlvg* mRNA Expression in Brachypterous *N. lugens* Adult Females After Third Instar Nymphs Fed on ABA-Treated Rice Plants

**Fig. 2.**
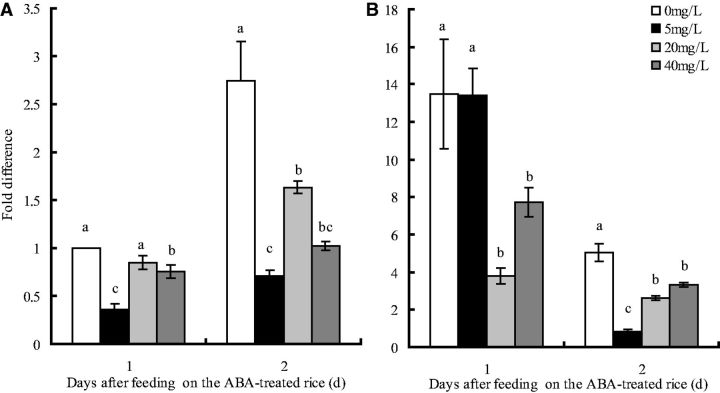
*Nlvg* mRNA expression in brachypterous *N. lugens* adult females prior to mating at 2 d after third instar *N. lugens* nymphs fed on exogenous ABA-treated rice plants for 1 or 2 d. *Nlvg* mRNA expression value in brachypterous *N. lugens* adult females of control [after third instar *N. lugens* nymphs fed on rice cultivar TN1 (without ABA spraying) for 1 day] was converted to 1. (A) Rice cultivar TN1 and (B) IR42. The means are shown ± SE, while different letters in the same line are significantly different at the *P* < 0.05 level. All values are normalized relative to *β* -actin transcript levels. Each treatment and control was repeated three times.

The three-way ANOVA showed that *Nlvg* mRNA expression in brachypterous *N. lugens* adult females varied significantly with rice cultivars ( *F* = 149.9, df = 1,32, *P* < 0.05; Fig. 2 ), days after third instar nymphs fed on ABA-treated rice plants ( *F* = 49.0, df = 1,32, *P* < 0.05; Fig. 2 ), and ABA concentrations ( *F* = 11.0, df = 3,32, *P* < 0.05; Fig. 2 ). Interaction effects between rice cultivars and days after fed on ABA-treated rice plants ( *F* = 79.2, df = 1,32, *P* < 0.05; Fig. 2 ), rice cultivars and ABA concentrations ( *F* = 8.4, df = 3,32, *P* < 0.05), and days after fed on ABA-treated rice plants and ABA concentrations ( *F* = 9.3, df = 3,32, *P* < 0.05), and among rice cultivars, days after fed on ABA-treated rice plants and ABA concentrations were also found ( *F* = 9.2, df = 3,32, *P* < 0.05; Fig. 2 ). Multiple comparisons of the means showed that after third instar *N. lugens* nymphs fed on ABA-treated rice plants and later fed on the control rice plants (without ABA spraying), *Nlvg* mRNA expression in brachypterous adult females decreased significantly compared with the control. *Nlvg* mRNA expression in brachypterous *N. lugens* adult females decreased by 0.63-, 0.15-, and 0.25-fold after fed on ABA-treated TN1 plants (5, 20, and 40 mg/liter) for 1 d, and decreased by 0.74-, 0.41-, and 0.63-fold after fed for 2 d, respectively. Similarly, *Nlvg* mRNA expression decreased by 0.72- and 0.43-fold after fed on ABA-treated IR42 plants (20 and 40 mg/liter, respectively) for 1 d and decreased by 0.83-, 0.45-, and 0.34-fold after fed on ABA-treated IR42 plants (5, 20, and 40 mg/liter, respectively) for 2 d.

#### Callose Contents in ABA-Treated Rice Plants Infested by Third Instar *N. lugens* Nymphs

**Fig. 3.**
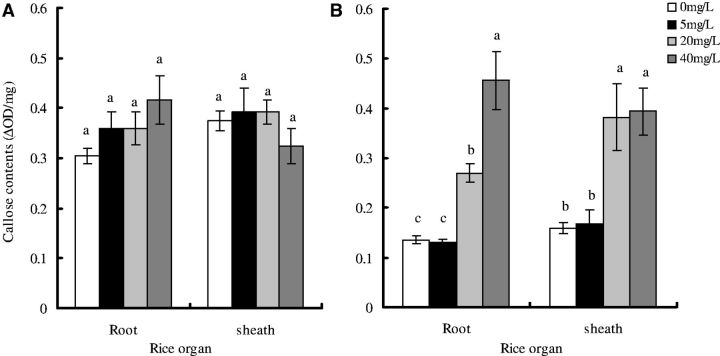
Effects of exogenous ABA treatments on callose contents in roots and sheaths of rice plants after infested by third instar *N. lugens* for 2 d. (A) Rice cultivar TN1 and (B) IR42. Data are presented as the means ± SE. Means followed by different letters are significantly different at the 5% level. Each treatment and control is repeated for four times.

The three-way ANOVA showed that callose contents in ABA-treated rice plants infested by third instar *N. lugens* nymphs for 2 d varied significantly with rice cultivars ( *F* = 30.9, df = 1,48, *P* < 0.05; Fig. 3 ), ABA concentrations ( *F* = 15.0, df = 3,48, *P* < 0.05), and the interaction effects between rice cultivars and ABA concentrations ( *F* = 14.1, df = 3,48, *P* < 0.05; Fig. 3 ). The rice organ (roots and sheaths; *F* = 1.5, df = 1,48, *P* > 0.05), and the interaction effects between rice cultivars and rice organ ( *F* = 0.1, df = 1,48, *P* > 0.05), and rice organ and ABA concentrations ( *F* = 2.8, df = 3,48, *P* > 0.05) and among rice cultivars, rice organ and ABA concentrations ( *F* = 0.5, df = 3,48, *P* > 0.05) did not vary significantly ( Fig. 3 ). Callose contents in ABA-treated rice plants infested by third instar *N. lugens* nymphs increased significantly, indicating that ABA induced the increase of callose contents in rice plants. Multiple comparisons of the means showed that, for IR42 plants, callose contents in roots increased significantly by 1.00- and 2.38-fold after third instar *N. lugens* nymphs fed on ABA-treated (20 and 40 mg/liter, respectively) rice plants compared with the control (without ABA treatments), and increased by 1.40- and 1.47-fold in sheaths, respectively. For TN1 plants, callose contents in roots and sheaths did not change significantly compared with the control.

## Discussion

Pieterse et al. (2001) found that plants were damaged by insects, and JA, SA, and ET were produced in the responses. Exogenous plant hormones, plant growth regulators, such as JA, SA, and ET, can influence the fecundity of insects, the effects of the exogenous plant hormones on insects mainly focused on JA, SA, and ET. For example, Omer et al. (2000) used exogenous applications of JA in cotton, which significantly affected the fecundity of the Pacific spider mite, the development rate and fecundity of grape phylloxera, *Viteus vitifoliae* . The application of JA also induces systemic defenses in tomato that reduces the effects of induced resistance in aphid feeding, development, reproduction, and mortality ( Cooper and Goggin 2005 ). In addition, Gong et al. (2010) found that exogenous JA-induced resistance in wolfberry shows important effects on the development and fecundity of the wolfberry *Aphis* sp., that the influence was related to JA concentration and that adult fecundity was reduced significantly. In recent years, many studies have found that ABA application also influences the physiology of plant. For example, Sandhu et al. (2010) found that exogenous application of ABA enhances the antioxidant capacity and increases the anthocyanin and phenolic content of muscadine grapes, and Leung and Giraudat (1998) had found that ABA promote seed maturation and germination. They serve as a signaling compound when plants are under stress including drought, high salinity, cold, and microbial infections. But rather the effects of ABA application on insects have received little attention. Our results showed that exogenous ABA-induced rice resistance to *N. lugens* significantly decreased the average injury scale of rice plants and had a negative impact on the *Nlvg* mRNA expression of *N. lugens* adult females. The decrease of *Nlvg* mRNA expression in *N. lugens* adult females may be attributed to the increasing resistance of rice plants after ABA treatments. Liu et al. (2012) demonstrated that the insecticides influenced the fecundity of *N. lugens* by affecting the *Nlvg* mRNA gene expression, from the relationship between the *Nlvg* mRNA gene expression and the fecundity of *N. lugens* . In addition, Liu et al. (2013) also found that potassium level in rice plants affected the *Nlvg* mRNA gene expression of *N. lugens* , and also affected the number of eggs of *N. lugens.* The expression of *Nlvg* is consistent with the eggs of *N. lugens* . So we can infer that the ABA application maybe decrease the fecundity of *N. lugens* . Mialoundama et al. (2009) showed that ABA acted as negative regulators of biotic stress. Whether the increase in rice plants resistance induced by exogenous ABA was due solely to endogenous ABA or to interactions with other plant hormones also requires further study.

Callose deposition can be induced by wounding, infection by pathogens, aluminum, ABA, and other physiological stresses ( Chen and Kim 2009 , You et al. 2010 ). For example, in some plant–pathogen interactions, ABA seems to exert a positive role in activating the plants’ own defense mechanisms, particularly by inducing the synthesis of callose, the main constituent of papollae, whose apposition in plant tissues represents an effective defense response at sites of attempted pathogen penetration ( Ton and Mauch-Mani 2004 , Flors et al. 2005 , Asselbergha and Hofte 2007 ). Recent studies have shown that exogenous application of ABA also induced significant plant resistance to tobacco necrosis virus ( Iriti and Faoro 2008 ), and the rise of ABA synthesis induced by chitosan plays an important role in enhancing callose deposition. In addition, Hao et al. (2008) indicated that *N. lugens* feeding induced callose deposition in the sieve tubes at the point where the stylet was inserted, and compact callose remained intact in the resistant plants, but the genes encoding *β* -1,3-glucanases were activated, causing unplugging of the sieve tube occlusions in susceptible plants. While Thaler and Bostock (2004) found that ABA-deficient plants had reduced resistance to the insect *Spodoptera exigua* . After ABA spraying, callose deposition in rice plants may change, which suggests that ABA may be related to plant resistance to insects by inducing the callose deposition, but callose contents appears to show no significant relationship with the concentration of ABA. From the relationship between callose contents of rice plants and *Nlvg* mRNA expression in *N. lugens* adult females, the decrease of *Nlvg* mRNA expression in *N. lugens* adult females may be partially attributed to the increase of callose contents of rice plants after ABA treatments, because in IR42 plants, callose contents increased significantly, however in TN1 plants, callose contents did not increase significantly. The mechanism in different resistant rice varieties needs further study.

This study demonstrated that exogenous ABA induced rice plant resistance and this induction has a significant effect on the *Nlvg* mRNA expression of *N. lugens* adult females, which is the important gene to affect the fecundity of brachypterous *N. lugens* adult females. These findings increase our knowledge concerning the effects of ABA-induced rice plant defenses on the phloem-feeding insects.
